# Whole-exome sequencing identified a missense mutation in *WFS1* causing low-frequency hearing loss: a case report

**DOI:** 10.1186/s12881-017-0511-7

**Published:** 2017-12-19

**Authors:** Hye Ji Choi, Joon Suk Lee, Seyoung Yu, Do Hyeon Cha, Heon Yung Gee, Jae Young Choi, Jong Dae Lee, Jinsei Jung

**Affiliations:** 1 Department of Otorhinolaryngology, Brain Korea 21 PLUS Project for Medical Sciences, Yonsei University College of Medicine, Seoul, 03722 South Korea; 2 Department of Pharmacology, Brain Korea 21 PLUS Project for Medical Sciences, Yonsei University College of Medicine, Seoul, 03722 South Korea; 3 Department of Otorhinolaryngology, Soonchunhyang University College of Medicine, Bucheon, South Korea; 4 Yonsei University College of Medicine, 134 Sinchon-dong, Seodaemun-gu, Seoul, 120-752 South Korea

**Keywords:** *WFS1*, Low-frequency hearing loss, Nonsyndromic hearing loss, Autosomal dominant

## Abstract

**Background:**

Low-frequency nonsyndromic hearing loss (LF-NSHL) is a rare, inherited disorder. Here, we report a family with LF-NSHL in whom a missense mutation was found in the Wolfram syndrome 1 (*WFS1*) gene.

**Case presentation:**

Family members underwent audiological and imaging evaluations, including pure tone audiometry and temporal bone computed tomography. Blood samples were collected from two affected and two unaffected subjects. To determine the genetic background of hearing loss in this family, genetic analysis was performed using whole-exome sequencing. Among 553 missense variants, c.2419A → C (p.Ser807Arg) in *WFS1* remained after filtering and inspection of whole-exome sequencing data. This missense mutation segregated with affected status and demonstrated an alteration to an evolutionarily conserved amino acid residue. Audiological evaluation of the affected subjects revealed nonprogressive LF-NSHL, with early onset at 10 years of age, but not to a profound level.

**Conclusion:**

This is the second report to describe a pathological mutation in *WFS1* among Korean patients and the second to describe the mutation in a different ethnic background. Given that the mutation was found in independent families, p.S807R possibly appears to be a “hot spot” in *WFS1*, which is associated with LF-NSHL.

## Background

Hearing loss is a common sensory disorder in humans and is caused by genetic mutations in more than 60% of cases. According to the Hereditary Hearing Loss Homepage (http://hereditaryhearingloss.org), autosomal dominant, nonsyndromic hearing loss is known to be associated with 59 genetic loci. Among these loci, which are designated DFNA (DFN = deafness; A = dominant), *DFNA6*, *DFNA14*, and *DFNA38* are associated with low-frequency nonsyndromic sensorineural hearing loss (LF-NSHL) and caused by a heterozygous mutation in the Wolfram syndrome 1 (*WFS1*) gene [1, 2].

*WFS1* is located on human chromosome 4p16.1, and the eight exons of *WFS1* encode a transmembrane protein. Exon 1 is noncoding, and exon 8 is the largest, containing 2.6 kb of DNA. Most mutations in *WFS1* have been identified in exon 8 and, moreover, in exons 3, 4, 5, and 6–8. The *WFS1* gene encodes wolframin, an 890-amino acid protein with an estimated molecular mass of 100 kDa. Wolframin is abundantly expressed in the pancreas, brain, heart, and muscle and is a hydrophobic and tetrameric protein with nine transmembrane domains and large hydrophilic regions at both ends [3, 4]. Mutations in *WFS1* are associated with autosomal recessive Wolfram syndrome and the autosomal dominant type of LF-NSHL (DFNA6/14/38). Wolfram syndrome has been identified in patients with diabetes insipidus, diabetes mellitus, optic atrophy, and hearing loss [5].

Using whole-exome sequencing (WES), we examined a Korean family (Yonsei University Hearing Loss [YUHL] 30) and identified a missense mutation, c.2419A → C (p.Ser807Arg), in the *WFS1* gene. In members of the pedigree, the mutation led to nonsyndromic autosomal dominant, symmetrically bilateral hearing loss at low-to-mid frequencies.

## Case presentation

**Fig. 1:**
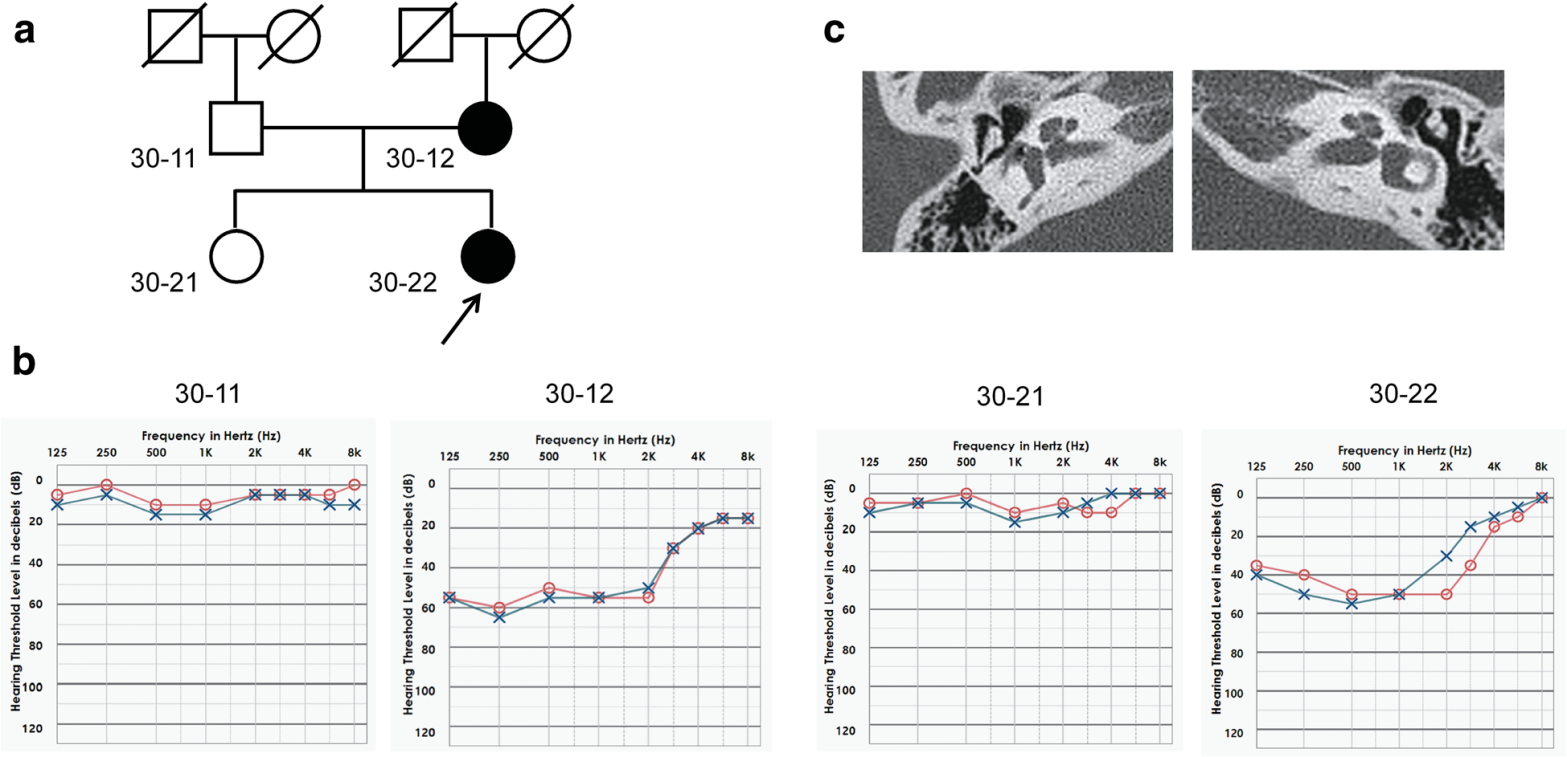
Pedigree and audiological evaluation. **a** Pedigree of the family (Yonsei University Hearing Loss [YUHL] 30 family) is shown. Affected individuals are denoted by gray symbols, males are denoted by squares, and females are denoted by circles. The affected proband (30–22) was a 14-year-old female with low-to-mid frequency nonsyndromic hearing loss. **b** Pure-tone audiometry of the subjects is depicted. Frequencies (Hz) were plotted on the x-axis and the auditory threshold in decibels (dB HL) was plotted on the y-axis. Red circles refer to the right ear, and blue crosses refer to the left ear

With approval from the Institutional Review Board of the Severance Hospital, Yonsei University (Seoul, South Korea) Health System (IRB#4–2015-0659), subjects with inherited hearing loss were enrolled in the YUHL cohort. After obtaining written informed consent, two affected and two unaffected subjects in the YUHL30 family were investigated. The pedigree of the YUHL30 family is shown in Fig. 1a. All affected subjects exhibited sensorineural hearing loss but no other syndromic phenotypes. The blood sugar levels of the subjects were within the normal range, and no subjects showed clinical symptoms suggesting diabetes mellitus or insipidus. In addition, the affected subjects had normal vision and no optic atrophy in ophthalmic evaluations. The proband 30–22 exhibited nonsyndromic autosomal dominant hearing loss at low-to-mid frequencies, of which the pure-tone threshold averages of 500, 1000, 2000, and 4000 Hz were 41 and 36 dB HL in the right and left ears, respectively (Fig. 1b). The hearing loss was symmetrically bilateral and was mild to moderate in severity. In speech audiometry, the word recognition score was 100% for both ears. In impedance audiometry, both drums were type A (compliance was 0.5 cm for both ears). In temporal bone CT scans, there were no inner ear anomalies or middle ear deformities (Fig. 1c). The mother of subject 30–22 (30–12) was also affected and exhibited a moderate level of bilateral sensorineural hearing loss at low-to-mid frequencies. The onset of hearing loss occurred at approximately 10 years of age in both subjects (30–22 and 30–12). While hearing function at frequencies between 2000 and 8000 Hz was well preserved, the hearing threshold was increased up to 50–60 dB HL at frequencies of 2000 Hz or less in the two affected subjects. Notably, it did not appear that hearing loss was progressive, since 30–12 (45 years of age) exhibited a degree and pattern of hearing loss similar to 30–22 (14 years of age).

**Fig. 2:**
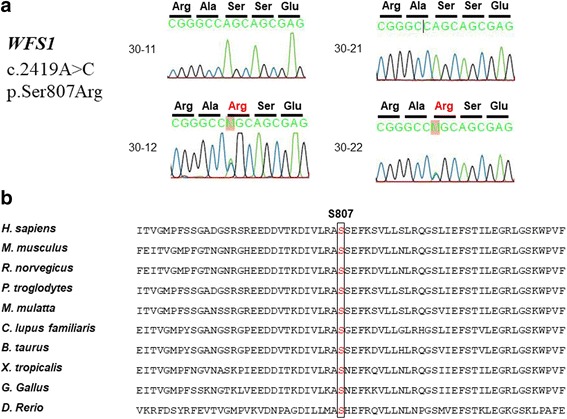
A mutation in *WFS1* identified using whole-exome sequencing. **a** Sanger sequencing traces of subjects 30–11, 30–12, 30–21, and 30–22. **b** Multiple sequence alignment of *WFS1* among different species. p.S807 is well preserved among various species

To identify the genetic cause of hearing loss in this family, WES was performed for 30–22, from whom DNA samples were available. DNA preparation, WES, sequence alignment, and variant calling were performed as previously reported [6]. Based on the pedigree and the fact that the affected individuals were a parent and her child, autosomal dominant hearing loss was assumed (Fig. 1a). There were 622 variants which were nonsynonymous or located in a splice site; a variant in *WFS1* (c.2419A → C; p.Ser807Arg) remained after filtering and inspection of WES data (Tables 1 and 2). This variant was a missense mutation and not found in public databases, such as dbSNP (http://www.ncbi.nlm.nih.gov/projects/SNP/) or gnomAD, for the affected status. Additionally, this mutation was associated with an alteration in an amino acid residue that has been well conserved throughout evolution from *Mus musculus*, *Gallus gallus*, *Xenopus tropicalis*, and *Danio rerio* (Table 2, Fig. 2a, b). Mutations in *WFS1* are known to cause autosomal dominant deafness (MIM 600965) [7]. The p.S807R mutation has been reported once in the United Kingdom but not in other counties, including Korea [7].

**Table 1:** Filtering process of whole exome sequencing analysis in this study

Patient (30–22)	
Total sequence reads	93,205,604
Matched Reads (percentage of reads)	91,298,272 (97.95%)
Total number of variants detected	187,202
Variants which are not common dbSNP138 (MAF > 1%) (A)	37,206
Variants which are not present in 32 control of healthy individuals	9487
Variants which are nonsynonymous or located in splice junction (B)	622
% B / A	1.67%
Located within splice site	31
Deletion/Insertion	33
Stop codon gained / Stop codon lost	5
Missense	553
SNPs after inspection of MAF < 0.05% & amino acid conservation	168
Located within splice site	7
Deletion/Insertion	3
Stop codon gained / Stop codon lost	5
Missense	153
Variant in 129 genes known to deafness genes	4
Sanger confirmation / Segregation	*2*
Causative mutation	*WFS1*

**Table 2:** Mutation candidates identified by whole exome sequencing

Gene Symbol	Individual	Sex	Nucleotide change^a^	Amino acid change	Exon (zygosity, segregation)	Amino acid sequence conservation^b^	dbSNP^c^	gnomAD^d^	PP2^e^	MT^f^	PROVEAN^g^	SIFT^h^
***WFS1***	30–22	F	c.2419A > C	p.Ser807Arg	8 (het, Mo)	*M. musculus*, *G. gallus*, X. tropicalis, *D. rerio*	ND	ND	Possibly Dam (0.890)	DC (0.999)	Del (−2.75)	Dam (0.012)

*Dam* damaging, *DC* disease causing, *Del* deleterious; het, heterozygous in affected individual, *MAF* minor allele frequency, *F* Female, *Mo* heterozygous mutation identified in mother, *ND* no data or DNA available, *SNP* single nucleotide polymorphism, *D. rerio Danio rerio*, *G. galllus Gallus gallus*, *M. musculus Mus musculus*, *X.tropicalis Xenopus tropicalis*

^a^Complementary DNA (cDNA) mutations are numbered according to human cDNA reference sequence NM_006005.3 (*WFS1*);

^b^Amino acid residue is continually conserved throughout evolution, including the species indicated;

^c^dbSNP database (http://www.ncbi.nlm.nih.gov/SNP);

^d^gnomAD browser (gnomad.broadinstitute.org/);

^e^PolyPhen-2 (PP2) prediction score (HumVar), ranges from 0 to 1.0 (0 = benign, 1.0 = probably damaging [http://genetics.bwh.harvard.edu/pph2/]);

^f^Mutation taster (http://www.mutationtaster.org/);

^g^Protein Variation Effect Analyzer (PROVEAN; http://provean.jcvi.org/index.php);

^h^Sorting Intolerant from Tolerant (SIFT; http://sift.jcvi.org/)

## Discussion and conclusions

In the present study, we used WES to successfully identify a mutation in *WFS1* causing the autosomal dominant type of sensorineural hearing loss.

To date, more than 250 mutations in the *WFS1* gene have been reported worldwide (http://www.hgmd.cf.ac.uk/ac/index.php). Mutations in the *WFS1* gene are responsible for both Wolfram syndrome and autosomal dominant, nonsyndromic hearing loss (DFNA6/14/38) [1, 8, 9]. Wolfram syndrome is an autosomal recessive type disorder associated with diabetes mellitus, diabetes insipidus, optic atrophy, and hearing loss. In nonsyndromic hearing loss, DFNA6/14/38 leads to LF-NSHL. According to the literature, there are two known genes responsible for low-frequency hearing loss: *DIAPH1* and *WFS1* [7]. Mutations in *DIAPH1* lead to progressive hearing loss and profound bilateral deafness involving all frequencies. However, mutations in *WFS1* cause progressive hearing loss, but only at low frequencies and not until profound hearing loss in the case of nonsyndromic hearing loss [10, 11]. In the present study, p.S807R was also associated with LF-NSHL, with moderate severity at 60 dB HL. This audiological feature is unique only in hearing loss associated with mutations in *WFS1*; thus, screening of *WFS1* is a reasonable first step in cases of suspected LF-NSHL without progression to a profound level.

Our description of the p.S807R (c.2419A → C) mutation in *WFS1* is the second report of a mutation associated with LF-NSHL in Korean patients; the first report was the p.V412A mutation in *WFS1* [12]. Although there are more than 250 mutations in *WFS1*, some rare mutations have been identified in multiple independent families. In addition, there are only two mutations that span two different ethnic backgrounds: European (p.A716T) and Japanese (p.E864K) [10]. p.S807R was previously reported in a family from the United Kingdom [8]; however, this study is the first to identify and report the mutation in an East-Asian individual. Interestingly, all mutations found in both ethnicities (European and East Asian, including p.A716T, p.E864K, and p.S807R) are located in exon 8, which encodes the C-terminal intracytoplasmic domain. This finding indicates that these mutations may be “hot spot” mutations in exon 8 that span different ethnicities. To convincely tell the mutation is a founder mutation (the result of admixture of two ethnics) or a hot spot, it would be helpful to to compare haplotypes of our patients and British patients.

The majority of mutations associated with LF-NSHL are, in fact, missense mutations located in exon 8, whereas those linked to Wolfram syndrome are frameshift and nonsense mutations [7, 8, 10]. The mutations responsible for LF-NSHL do not inactivate *WFS1* and are presumed to have a dominant-negative effect on the normal *WFS1* protein, consistent with an autosomal dominant type of inheritance. Because the S807 residue is located in the C-terminal domain, the missense mutation at p.S807R affects only a limited number of functions. Although the physiological role of *WFS1* in the inner ear remains unknown, mutations, such as p.S807R, only partially disrupt the function of *WFS1*; thus, hearing loss may not necessarily progress to profound deafness.

Mutations in *WFS1* are associated with LF-NSHL. Given that p.S807R was previously found in individuals of European ethnicity, p.S807R may be a hot-spot mutation in *WFS1*. Because the majority of mutations in *WFS1* associated with LF-NSHL are located in exon 8, screening exon 8 should be considered first in genetic analyses of patients with LF-NSHL.

## Abbreviations

LF-NSHL: Low-frequency nonsyndromic hearing loss
WES: Whole-exome sequencing
WFS1: Wolfram syndrome 1
